# Protometabolism as out-of-equilibrium chemistry

**DOI:** 10.1098/rsta.2020.0423

**Published:** 2022-07-11

**Authors:** Serge Nader, Lorenzo Sebastianelli, Sheref S. Mansy

**Affiliations:** Department of Chemistry, University of Alberta, 11227 Saskatchewan Drive, Edmonton, AB, Canada T6G 2G2

**Keywords:** protometabolism, out-of-equilibrium, dissipative systems, origins of life, prebiotic chemistry

## Abstract

It is common to compare life with machines. Both consume fuel and release waste to run. In biology, the engine that drives the living system is referred to as metabolism. However, attempts at deciphering the origins of metabolism do not focus on this energetic relationship that sustains life but rather concentrate on nonenzymatic reactions that produce all the intermediates of an extant metabolic pathway. Such an approach is akin to studying the molecules produced from the burning of coal instead of deciphering how the released energy drives the movement of pistons and ultimately the train when investigating the mechanisms behind locomotion. Theories that do explicitly invoke geological chemical gradients to drive metabolism most frequently feature hydrothermal vent conditions, but hydrothermal vents are not the only regions of the early Earth that could have provided the fuel necessary to sustain the Earth's first (proto)cells. Here, we give examples of prior reports on protometabolism and highlight how more recent investigations of out-of-equilibrium systems may point to alternative scenarios more consistent with the majority of prebiotic chemistry data accumulated thus far.

This article is part of the theme issue ‘Emergent phenomena in complex physical and socio-technical systems: from cells to societies’.

## 1. Protometabolism

What makes chemistry metabolic is the ability to sustain a living cell, which means that chemistry prior to the emergence of life cannot be described as metabolic. Nevertheless, extant metabolic chemistry must have had progenitors, and it is this set of chemical reactions that existed before the transition to biology that is frequently referred to as protometabolism. However, the term has been used loosely, and we would argue in a way that clouds our understanding of the emergence of life. The chemistry that sustains life is complex with many pathways and components integrated together within a highly organized compartment that defies thermodynamic equilibrium. Narrow investigations of individual synthetic pathways in isolation and in absence of a compartment will likely be less informative than, as Ruiz-Mirazo *et al.* [1,2] argue, systems level approaches that search for commonalities and synergies between chemistries and phases that could help to explain why life is as it is today.

Extant metabolism consists of both catabolic and anabolic reactions, i.e. the breakdown and synthesis of molecules, respectively. The exergonic chemistry of catabolism drives the endergonic reactions of anabolism that are needed to sustain the cell. But, this is not a one-way process where anabolism relies on catabolism alone. The products of anabolism are needed to mediate and regulate catabolism. The current search for separate prebiotic analogues of extant anabolic and catabolic chemistries largely does not address this critical feature of biology, which is how prebiotic systems chemistry gave rise to a mixture of reactions that tie together in a way that defies equilibrium. Such primordial reaction networks that join endergonic with exergonic pathways, ideally through common currencies, as discussed below, would in our estimation be better described as protometabolic in comparison to narrower investigations of nonenzymatic mimics of extant catabolism or anabolism in isolation.

## 2. Prebiotic analogues of anabolism

Much effort has centred on prebiotic mimics of anabolism whereby plausible routes towards the synthesis of the building blocks of life are deciphered. What has been more contentious is whether these prebiotic reaction pathways resembled modern-day metabolism or were often distinct, reflecting the different constraints imposed by an ancient, prebiotic environment [3–6]. Both arguments invoke the ability of enzymes to facilitate but not invent chemistry, and both tend to rely on laboratory approximations of reactions under thermodynamic, as opposed to kinetic, control. Proponents of prebiotic chemistry unconstrained by extant biology find it unsurprising that biological reactions can be run nonenzymatically, particularly when subjected to extremes in temperature or pH. Activation energy barriers can be traversed without enzymes, so it would be more surprising if the reaction could not proceed at all. Therefore, observing an uncontrolled nonenzymatic analogue of extant metabolism may tell us little about the role of such chemistry in the origins of life, particularly if metabolism requires regulatory processes that are not easily obtainable without enzymes. Conversely, if a reaction can run nonenzymatically, why would it not? A functioning reaction cycle from which the building blocks of life can be derived is attractive as the emergence of modern-day metabolism could be envisaged to proceed by the stepwise addition of enzymes [7,8]. As with most arguments, reality likely does not fit cleanly into a single category. Just as the RNA world hypothesis gives the most compelling explanation for the rise of Darwinian evolution, it is naive to think that life began with RNA alone. Similarly, while the abundance of biological molecules that can be synthesized from cyanide prebiotically [9] cannot be ignored, it stands to reason that at least some prebiotic reactions were similar to contemporary biological analogues [10].

Although differences in opinion are healthy, particularly when subjected to experimental evaluation, opinions that lead to the imposition of poorly supported rigid constraints are unhelpful. Since we do not know the conditions that gave rise to life, we are left with the realities of chemical reactivity and geological plausibility. A path between nonenzymatic prebiotic chemistry that resembles extant biological pathways and the last universal common ancestor (LUCA) is easy to imagine, which leads many to confidently extrapolate back in time from our best guesses as to what LUCA looked like [11]. However, if such an approach is taken at the exclusion of a broader investigation of what prebiotically plausible molecules give rise to, then we are likely to be led astray. As others have argued before, the early steps leading to life may have been erased by time and thus cannot be easily perceived from extant artefacts [4,9]. Phylogeny can tell us much about the early evolution of life, but it is not logical to allow the genetics of a highly complex organism with 300–400 genes [12,13] dictate how chemistry prior to genetics must have proceeded. Ultimately, a thorough testing of different chemical scenarios congruent with geology is needed so that experimental data and hypotheses can be compared and evaluated [14].

Regardless of the vantage point, what works on prebiotic analogues of anabolism have in common is an attempt to understand how larger biological molecules could have been synthesized prebiotically from smaller, abundant chemical resources. Although there were early, important studies on the prebiotic synthesis of biological molecules [15,16], modern work applies knowledge of synthetic organic chemistry to reactions in water or at the air–water interface [17], under prebiotically plausible conditions. As CO_2_ was more abundant on the prebiotic Earth, and as the Wood–Ljungdahl pathway (or the reductive acetyl-coenzyme A pathway) has been hypothesized to have been exploited by LUCA, CO_2_ often features as a carbon source for the synthesis of more complex molecules. Metallic iron or precipitates of metal ions under a variety of conditions have been found to reduce CO_2_ to formate, methanol, methane, acetate and pyruvate [18–21]. Metal ions are further invoked for the catalysis of several steps of an abiotic reductive citric acid cycle, which can be coupled to the synthesis of alanine [22]. Alternatively, glycolate can be synthesized from CO_2_ and in the presence of UV light and sulfite without the participation of metals [23]. Further irradiation of the glycolate with sulfite produces several components of the citric acid cycle, including citrate, malate and succinate [23]. Metal-free reactions between pyruvate and glyoxylate can also lead to a prebiotic analogue of the reductive citric acid cycle and produce amino acids via transamination with glycine [24]. Conversely, if one considers the rich chemistry of the planetary abundant cyanide [25–27], particularly in the presence of UV light, metal ions and reductants such as sulfite, then over half of the 20 amino acids [28,29], pyrimidine and purine ribonucleotides [30–33] and precursors to lipids [29] can all be built by photoreductive homologation pathways orthogonal to that of extant biology. Although we favour the robust non-biological-like routes to the synthesis of the building blocks of life, and it is important to directly compare published data from different perspectives [3], the intent here is not to promote or diminish one perspective but to highlight that several paths have been published for the synthesis of the building blocks and that these pathways often give rise to and consume pyruvate and other *α*-ketoacids.

## 3. Prebiotic analogues of catabolism

Surprisingly, less effort has been expended in investigating prebiotic mimics of catabolism. Metabolic intermediates, such as glucose-6-phosphate, have been shown to break down to pyruvate in the presence of Fe^2+^ at elevated temperatures in a manner reminiscent of glycolysis [34]. Similar conditions lead to both the synthesis and degradation of the intermediates of the citric acid cycle when starting from pyruvate and glyoxylate [35]. The reaction pathway can be exploited to synthesize amino acids if hydroxylamine and metallic iron are added [35]. Non-metal-dependent catabolic-like pathways have also been described. An analogue of glycolysis starting from the simple sugar glyceraldehyde gives rise to a series of reactions that generate phosphoenolpyruvate and pyruvate when fed with cyanide, the phosphorylating agent diamidophosphate [36] and glycoaldehyde [37]. Additionally, two interconnecting reaction networks, referred to as the 4-hydroxy-2-ketoglutarate and malonate cycles, that function as a type of prebiotic analogue of the citric acid cycle are sustained by feeding with pyruvate, glyoxylate and hydrogen peroxide [38]. Here, reaction intermediates can be syphoned off to synthesize aspartate in the presence of ammonia [38]. As with the prebiotic analogues of anabolic reactions, the published prebiotic analogues of catabolic networks frequently feature pyruvate and other *α*-ketoacids.

**Figure 1.**
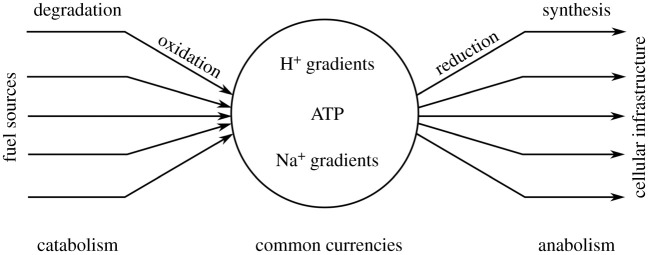
Extant metabolism works, in part, by funnelling the energy released from the oxidative degradation of varied fuel sources into common currencies, which are then used to drive the energetically costly reductive synthetic processes needed to sustain the cell.

The aforementioned work demonstrates what types of reactions and molecules were potentially chemically accessible on the prebiotic Earth. Such research is critically important, because knowing what was present addresses what types of protocells could have formed. However, the ingredients of life alone are not enough to make a living cell. If that were so, then we would be able to take apart and put back together a living cell. Further, metabolism is more dissimilar to laboratory-based synthesis than is often appreciated. Scientists are skilled at manipulating thermodynamics and reactivity to push reactions forward, but metabolism does not solely rely on such direct manipulations of chemistry. Instead, metabolism elegantly ties the two branches (i.e. anabolic and catabolic) together through an intermediary process that deposits, stores and spends energy to sustain a vast array of endergonic chemistry (figure 1). That is, life-as-we-know-it plugs into a fuel source with a wire of molecules that consecutively transfers electrons [39]. The molecules that engage in electron transfer most commonly include metallocofactors, ribodinucleotides and quinones. The fuel can be reduced organic molecules, such as sugars and lipids, or inorganic molecules, such as molecular hydrogen, hydrogen sulfide and ferrous ions. The thermodynamically favourable flux of electrons through the cell is coupled to processes that store the released energy into common currencies. This is a key invention of biology [39] and is distinct from the more direct coupling that is typically used in prebiotic chemical studies. In biology, the common currencies consist of H^+^ gradients, Na^+^ gradients and adenosine triphosphate, which are used by the cell to pay for the costly work of manufacturing and repairing cellular infrastructure [39]. What is unclear is whether such regulated fluxes of energy that are used to keep cellular systems out-of-equilibrium emerged early or were a later invention.

**Figure 2.**
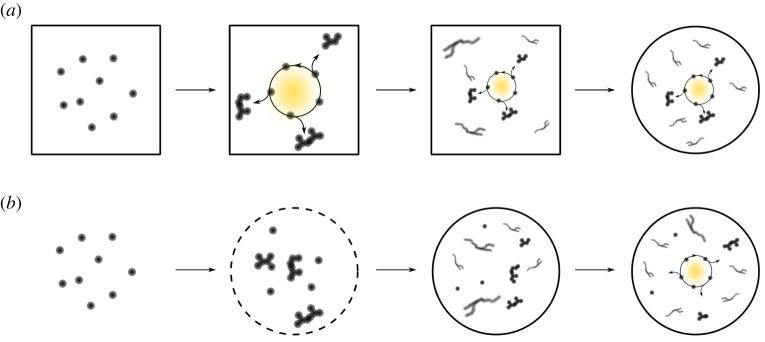
Comparing timelines of early life innovations. (*a*) A generic representation of hydrothermal vent scenarios where a constant influx of energy and matter through porous rock sustains prebiotic analogues of metabolism. Over time, the system evolves, generating biopolymers and other molecular machinery before an escape event that leads to encapsulation within a lipid vesicle. (*b*) A generic representation of the surface of the early Earth where building blocks assemble preferentially within lipid vesicles. This spatial isolation imposes a selective pressure that gives rise to protocells supported by an internal chemistry. (Online version in colour.)

The concept of life being a by-product of dissipating energy gradients is not new [40]. Others have eloquently discussed the potential chemiosmotic [41] origins of life, and how geological gradients could have given rise to the Earth's first cells [42]. Broadly speaking, such hypotheses tend to place the origins of life at hydrothermal vents, which is in contrast to more genetic centric work, such as the RNA world hypothesis, which has favoured surface conditions. Although both perspectives may focus on syntheses starting from different carbon sources (carbon dioxide versus hydrogen cyanide) and with a bigger or smaller role of metal ions, in a narrow sense, there is commonality in that both frequently seek to delineate how the building blocks of life were made. The deeper difference between the two perspectives concerns the placement of metabolism within a timeline encompassing the emergence of the Earth's first cells (figure 2). In other words, were the building blocks of life synthesized by a protometabolic system prior to the emergence of a protocell (figure 2*a*) or did the building blocks accumulate by distinct chemistry before the advent of protometabolism (figure 2*b*)? Thus far, much more experimental work has been carried out on chemistry congruent with surface conditions where the building blocks could have accumulated [3,43–45]; however, studies have recently begun to investigate the plausibility of hydrothermal vent scenarios guided by the presumption of an earlier appearance of protometabolism [20,46,47]. While we appreciate some aspects of the hydrothermal vent theories, such as the role of metal ions, including iron-sulfur clusters [48,49], and the emphasis on energetics, hydrothermal vents are not the only places capable of providing an energy source.

## 4. Non-hydrothermal vent, out-of-equilibrium processes

**Figure 3.**
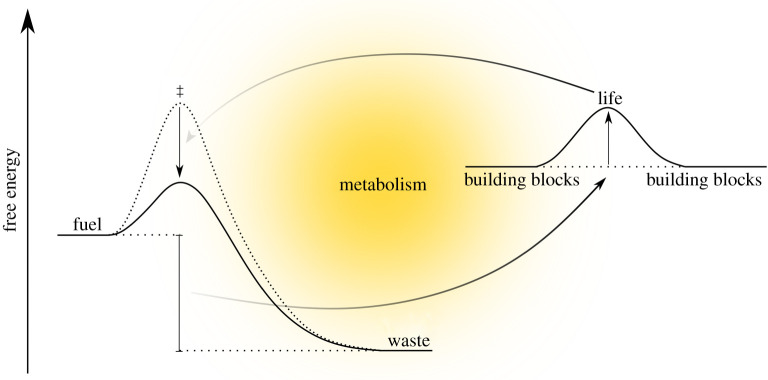
Living systems use metabolism to exploit the free energy released from the degradation of fuel to maintain their out-of-equilibrium state. As life is a chemical unit capable of catalysing the degradation of fuel and is capable of proliferation, a feedback loop is established between the fuel containing environment and the living organism. (Online version in colour.)

Living cells are out-of-equilibrium chemical systems that harness the dissipation of fuel sources to maintain their highly ordered state (figure 3). The persistence of these metastable, out-of-equilibrium arrangements is also known as dynamic kinetic stability [50–52], which is at times additionally referred to as dynamic self-assembly when describing chemical systems without biological components [53]. Attempts to construct chemical systems that imitate features of life tend to focus on mimicking three different facets of life-as-we-know-it as opposed to building an integrated protocell, including (i) error-prone replication capable of Darwinian evolution, (ii) metabolic-like chemistry that sustains an overall dissipative system with a fuel source and (iii) compartmentalization to separate the chemical system from the environment. Although the assimilation of all three within a protocell is presumably needed to generate a highly adaptable system capable of surviving environmental fluctuations, more narrow investigations have already begun to reveal how mechanisms, such as autocatalysis, can give rise to characteristics that imitate some features of life. Importantly, these studies directly link thermodynamics, kinetics, competition and selection in a way that is absent in discussions of the energetic fluxes present at hydrothermal vents.

What these studies typically have in common is either the presence of autocatalysis or dependence on reactions that proceed along different paths in the forward and reverse directions. Autocatalysis is simply when the product of a reaction catalyses its own formation [54,55] and represents an efficient means of funnelling reactions down a restricted path towards a common product. A well-known example is the ability of the Soai reaction to resolve a nearly racemic mixture to enantiopurity, perhaps revealing the types of mechanisms that led to life being largely dependent on homochirality [55]. However, the examples typically encountered in origins of life research are more complex, rely on a phase transition of some type and focus on replication. For example, Luisi and co-workers [56] demonstrated how the hydrolysis of ethyl caprylate produces ethanol and the fatty acid caprylate. When sufficient fatty acid is produced, the caprylate assembles into micelles that then catalyse the formation of more fatty acid [56], although later computational work suggests that the mechanism is more complex [54,57,58]. Here, it is not solely the formation of a molecule, the fatty acid, that is critical, but that this molecule is able to self-assemble into a higher order aggregate structure that then pulls the system away from equilibrium in addition to acting as a catalyst for the synthesis of more fatty acid. This type of A + B ⇌ C → D, where D is an aggregate, or new phase, composed of C is common.

**Figure 4.**
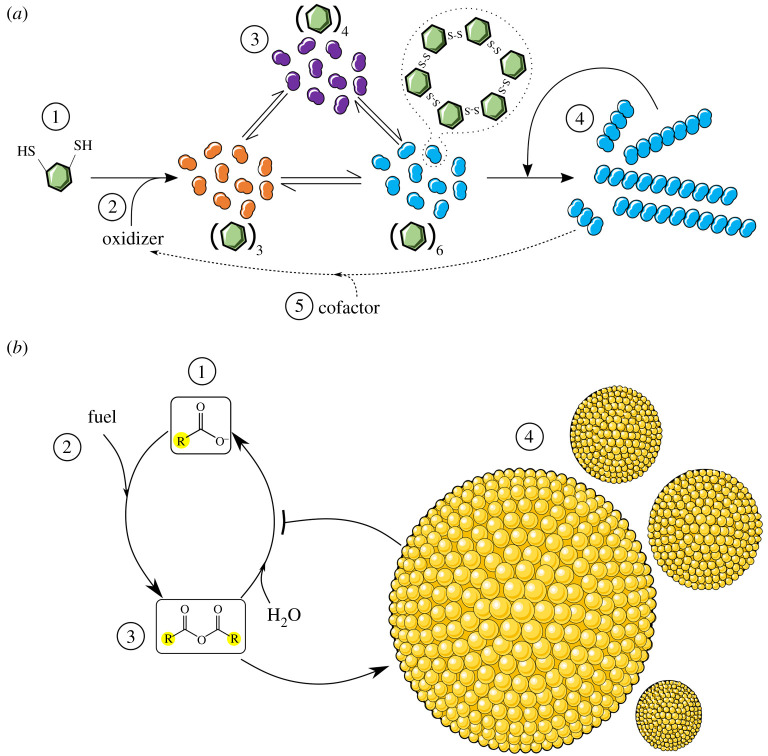
Self-assembly pulls systems out-of-equilibrium. (*a*) Autocatalysis. Building blocks (1), e.g. thiol containing peptides, react with a fuel molecule or oxidant (2) to form a dynamic combinatorial library (3). One molecular member of the library can self-assemble (4), thus pulling the system out-of-equilibrium. The self-assembled state (4) itself, or after mechanical breakage, can catalyse the formation of more of the self-assembled state. Further, new properties of the self-assembled state, either intrinsically or by uptake of cofactors from the environment, may emerge which facilitate the productive consumption of building blocks (5). (*b*) Differential formation and degradation pathways. A mixture of soluble building blocks (1), e.g. fatty acids, react with fuel molecules (2), such as a carbodiimide, to generate products of decreased solubility (3) that can be degraded back to the building block through hydrolysis. The self-assembly of a subset of products (4) inhibits the back reaction, thereby pulling the system away from equilibrium and sustaining an assembly of unstable molecules over time. (Online version in colour.)

More recent examples [59] with modified peptides instead of fatty acids exploit the ability of some of the intermediate products to aggregate into fibres (figure 4*a*). In one example, peptides are modified with a benzenedithiol group that upon oxidation forms an array of disulfide macrocycles in which one specific type, the hexameric form, assembles into fibres [60,61]. In this case, the fibres represent the newly formed phase similar to the micelle example above. If none of the disulfide macrocycles formed were capable of phase separation, then the final pool of molecules would reflect the free energy of the products and their placement within the free energy landscape [62]. Conversely, when one molecule is capable of self-assembly, the gained free energy favours the accumulation of the self-assembled state at the expense of the non-assembling competitors [60,63]. The dynamics of similar systems has been studied [64], including systems with aggregates composed of prebiotic precursors of RNA [65,66]. However, the described peptide version can also be mechanically broken, leading to a form of replication whereby the fibre fragments seed the growth of more fibres in a way similar to that seen with crushed crystals [61].

The key to ensuring a continuous out-of-equilibrium state is the coupling of the system to its energy source. Therefore, the building blocks cannot be completely consumed and the final self-assembled state itself should not inhibit exponential self-replication [67]. Serial transfer, whereby a portion of the self-replicating set is introduced to a new environment with replenished fuel sources, can be used to overcome equilibrium [68]. Perhaps more elegantly, the mechanically induced breakage of fibres described above additionally helps to avoid reaching equilibrium, because the broken fibres increase the number of available self-replicating template sites [61]. That is, the change in copy number of the self-replicator further pulls the system towards dynamic kinetic stability instead of thermodynamic equilibrium [50–52]. One objective of those working on such systems is to allow for open-ended evolution in a way similar but not limited to Red Queen coevolution [69,70] whereby a self-replicating entity partially, or wholly, displaces the free energy landscape of a system. As a consequence, other self-replicating entities present within the ecosystem must engage in reciprocal adaptive interactions. The resulting ecosystem has the potential to overcome Eigen's paradox, which relates the complexity of the system to copying fidelity [71], because interconnected catalytic hypercycles can function as a dynamic community ensuring accuracy in self-replication, in a way similar to Eigen's quasi-species model [72].

The goal in research on self-replicators is often to identify replicators that acquire metabolism by catalysing chemical reactions within the environment in a way that promotes their own replication. In impressive work by Otto and co-workers [73], peptide fibres were found to recruit chromophore cofactors from the environment, which led to the photooxidation of the building blocks. As oxidation of the building blocks is necessary to form the disulfide linked macrocycles necessary for subsequent self-assembly into fibres, a positive feedback loop emerged from the chemical system that allowed the self-replicator to better harness the surrounding environment. Significantly, such feedback loops can also emerge from the intrinsic properties of the fibres themselves through interactions with the environment without the need of cofactors [74].

## 5. Non-autocatalytic out-of-equilibria

It is additionally possible to maintain the existence of a thermodynamically unfavourable state without autocatalysis if the product of the pathway is formed and degraded by different paths [75]. In this case, the depletion of the fuel leads to the disassembly of the non-kinetically trapped aggregate. Such examples typically exploit phase separation, with the formation of actin filaments and microtubules representing biological examples [75]. An instructive case is that of the carbodiimide mediated formation of fatty acid anhydrides that then phase separate into oil droplets that are more resistant to hydrolysis back to the fatty acid building block [76] (figure 4*b*). If the starting pool of building blocks consists of fatty acids of different lengths, then the fatty anhydrides of the longer chain fatty acids preferentially form oil droplets, meaning that the major products consist of the longer chain fatty anhydrides. As expected, the preferential formation of one subset of fatty anhydrides is accentuated by repeated refuelling, particularly when performed in batch additions as opposed to continuous feeding. Therefore, even without autocatalysis, competition can emerge between different chemical systems, with one, out-of-equilibrium subset persisting and out-competing competitors over time [76]. It is interesting to note that older examples of chemical systems persisting in the presence of separate chemical reactions that feed and degrade fatty acid vesicles were reported in support of the importance of autopoiesis in the origins of life [77].

## 6. Conclusion

We have not attempted to go through all the examples but rather to point out that dissipative systems that mimic some critical features of life can emerge in scenarios that do not depend on hydrothermal vent conditions [78–81]. Energy is not a unique property of hydrothermal vents. The Sun is a clear example of a prebiotic energy source that would have been inaccessible in the deep sea [3,17,44], and chemical energy sources, e.g. isonitriles, likely drove prebiotic chemistry forward [82]. Work on non-hydrothermal, out-of-equilibrium systems thus far has made impressive progress, but none of these previous studies attempted to work within prebiotically plausible conditions and rarely made use of molecules that were clearly on a path from prebiotic chemistry to life-as-we-know-it, with a possible exception being the formose reaction [83], although not universally accepted [84]. If prebiotically plausible dissipative systems were experimentally demonstrated that clearly tied into a protocellular architecture in a way that helped explain extant biology, then the case for one prebiotic scenario over another would be strengthened. Such a system may look quite different from the directly coupled examples developed thus far, because the way in which biology exploits the dissipation of a fuel source to drive cellular organization and function is considerably more complex. It is, therefore, fair to ask if extant metabolic-like chemistry emerged early or was a later invention.

To help gain insight into this problem, we believe that more work is needed that integrates multiple components and pathways together into protocellular compartments. That is, we should take what has been learned from out-of-equilibrium, dissipative chemical systems and apply these principles to the construction of fuel-driven, prebiotically plausible protocells. The lack of use of vesicles in studies on protometabolism is surprising, as the simple presence of a membrane alters chemistry, facilitating peptide synthesis [85,86], competition [86,87] and provides for ways to tie chemistry to the survivability of the protocell [88]. There are already some clues regarding how catabolic and anabolic chemistry could have been coupled. The prebiotic synthesis of iron–sulfur clusters has been demonstrated [89], and iron–sulfur peptides can engage in electron transfer reactions that lead to the generation of a proton gradient across diacyl phospholipid membranes [90]. Further, prebiotic fuel sources, such as α-ketoacids, can initiate electron transfer reactions in a way that resembles what is found in biology [91]. It seems that several pieces of the puzzle are there, ready to be put in place, with the striking exception of nucleic acids. Although the challenge is great, we suspect that many critical insights will come from a more concerted effort to investigate how prebiotically plausible chemistry could have fuelled early protocells. Regardless of the outcome, the data will likely help settle some of the ongoing debates regarding the role of (proto)metabolism in the origins of life.

## Data Availability

This article has no additional data.

## References

[1] Ruiz-Mirazo K. 2020 Boundary versus enabling conditions for the origins of life. Phys. Life Rev. **34–35**, 96-98. (10.1016/j.plrev.2020.06.001) 32536537

[2] Ruiz-Mirazo K, Briones C, de la Escosura A. 2014 Prebiotic systems chemistry: new perspectives for the origins of life. Chem. Rev. **114**, 285-366. (10.1021/cr2004844) 24171674

[3] Green NJ, Xu J, Sutherland JD. 2021 Illuminating life's origins: UV photochemistry in abiotic synthesis of biomolecules. J. Am. Chem. Soc. **143**, 7219-7236. (10.1021/jacs.1c01839) 33880920 PMC8240947

[4] Krishnamurthy R. 2018 Life's biological chemistry: a destiny or destination starting from prebiotic chemistry? Chem. Eur. J. **24**, 16 708-16 715. (10.1002/chem.201801847) 29870593

[5] Lazcano A. 2009 Complexity, self-organization and the origin of life: the happy liaison? In Origins of life: self-organization and/or biological evolution? [internet], pp. 13-22. Paris, France: EDP Sciences. See http://www.origins-and-evolution.org/10.1051/orvie/2009002.

[6] Muchowska KB, Varma SJ, Moran J. 2020 Nonenzymatic metabolic reactions and life's origins. Chem. Rev. **120**, 7708-7744. (10.1021/acs.chemrev.0c00191) 32687326

[7] Lazcano A, Miller SL. 1999 On the origin of metabolic pathways. J. Mol. Evol. **49**, 424-431. (10.1007/PL00006565) 10486000

[8] Ralser M. 2014 The RNA world and the origin of metabolic enzymes. Biochem. Soc. Trans. **42**, 985-988. (10.1042/BST20140132) 25109990 PMC4128644

[9] Wu L-F, Sutherland JD. 2019 Provisioning the origin and early evolution of life. Bayley H, editor. Emerg. Top. Life Sci. **3**, 459-468. (10.1042/ETLS20190011) 32002470 PMC6992421

[10] Peretó J. 2012 Out of fuzzy chemistry: from prebiotic chemistry to metabolic networks. Chem. Soc. Rev. **41**, 5394. (10.1039/c2cs35054h) 22508108

[11] Weiss MC, Preiner M, Xavier JC, Zimorski V, Martin WF. 2018 The last universal common ancestor between ancient Earth chemistry and the onset of genetics. Achtman M, editor. PLoS Genet. **14**, e1007518. (10.1371/journal.pgen.1007518) 30114187 PMC6095482

[12] Gil R, Silva FJ, Peretó J, Moya A. 2004 Determination of the core of a minimal bacterial gene set. MMBR **68**, 518-537. (10.1128/MMBR.68.3.518-537.2004) 15353568 PMC515251

[13] Weiss MC, Sousa FL, Mrnjavac N, Neukirchen S, Roettger M, Nelson-Sathi S, Martin WF. 2016 The physiology and habitat of the last universal common ancestor. Nat. Microbiol. **1**, 16116. (10.1038/nmicrobiol.2016.116) 27562259

[14] Ruiz-Mirazo K. 2019 Reaction: a plea for hypothesis-driven research in prebiotic systems chemistry. Chem **5**, 1920-1922. (10.1016/j.chempr.2019.06.009)

[15] Miller SL, Urey HC. 1959 Organic compound synthesis on the primitive earth: several questions about the origin of life have been answered, but much remains to be studied. Science **130**, 245-251. (10.1126/science.130.3370.245) 13668555

[16] Wöhler F. 1828 Ueber künstliche Bildung des Harnstoffs. Ann. Phys. Chem. **88**, 253-256. (10.1002/andp.18280880206)

[17] Deal AM, Rapf RJ, Vaida V. 2021 Water–air interfaces as environments to address the water paradox in prebiotic chemistry: a physical chemistry perspective. J. Phys. Chem. A **125**, 4929-4942. (10.1021/acs.jpca.1c02864) 33979519

[18] Cody GD. 2000 Primordial carbonylated iron-sulfur compounds and the synthesis of pyruvate. Science **289**, 1337-1340. (10.1126/science.289.5483.1337) 10958777

[19] Horita J. 1999 Abiogenic methane formation and isotopic fractionation under hydrothermal conditions. Science **285**, 1055-1057. (10.1126/science.285.5430.1055) 10446049

[20] Hudson R et al. 2020 CO_2_ reduction driven by a pH gradient. Proc. Natl Acad. Sci. USA **117**, 22 873-22 879. (10.1073/pnas.2002659117) PMC7502746 32900930

[21] Preiner M et al. 2020 A hydrogen-dependent geochemical analogue of primordial carbon and energy metabolism. Nat. Ecol. Evol. **4**, 534-542. (10.1038/s41559-020-1125-6) 32123322

[22] Muchowska KB, Varma SJ, Chevallot-Beroux E, Lethuillier-Karl L, Li G, Moran J. 2017 Metals promote sequences of the reverse Krebs cycle. Nat. Ecol. Evol. **1**, 1716-1721. (10.1038/s41559-017-0311-7) 28970480 PMC5659384

[23] Liu Z, Wu L-F, Kufner C, Sasselov DD, Fischer W, Sutherland J. 2021 Prebiotic Photoredox Synthesis from Carbon Dioxide and Sulfite [Internet]. 2021 Feb [cited 2021 May 30]. See https://chemrxiv.org/articles/preprint/Prebiotic_Photoredox_Synthesis_from_Carbon_Dioxide_and_Sulfite/13692772/1. 10.1038/s41557-021-00789-w PMC7611910 34635812

[24] Stubbs RT, Yadav M, Krishnamurthy R, Springsteen G. 2020 A plausible metal-free ancestral analogue of the Krebs cycle composed entirely of α-ketoacids. Nat. Chem. **12**, 1016-1022. (10.1038/s41557-020-00560-7) 33046840 PMC8570912

[25] Ferus M, Kubelík P, Knížek A, Pastorek A, Sutherland J, Civiš S. 2017 High energy radical chemistry formation of HCN-rich atmospheres on early earth. Sci. Rep. **7**, 6275. (10.1038/s41598-017-06489-1) 28740207 PMC5524942

[26] Miyakawa S, James Cleaves H, Miller SL. 2002 The cold origin of life: A. implications based on the hydrolytic stabilities of hydrogen cyanide and formamide. Orig. Life Evol. Biosph. **32**, 195-208. (10.1023/A:1016514305984) 12227424

[27] Smith KE, House CH, Arevalo RD, Dworkin JP, Callahan MP. 2019 Organometallic compounds as carriers of extraterrestrial cyanide in primitive meteorites. Nat. Commun. **10**, 2777. (10.1038/s41467-019-10866-x) 31239434 PMC6592946

[28] Foden CS, Islam S, Fernández-García C, Maugeri L, Sheppard TD, Powner MW. 2020 Prebiotic synthesis of cysteine peptides that catalyze peptide ligation in neutral water. Science **370**, 865-869. (10.1126/science.abd5680) 33184216

[29] Patel BH, Percivalle C, Ritson DJ, Duffy CD, Sutherland JD. 2015 Common origins of RNA, protein and lipid precursors in a cyanosulfidic protometabolism. Nat. Chem. **7**, 301-307. (10.1038/nchem.2202) 25803468 PMC4568310

[30] Becker S, Thoma I, Deutsch A, Gehrke T, Mayer P, Zipse H, Carell T. 2016 A high-yielding, strictly regioselective prebiotic purine nucleoside formation pathway. Science **352**, 833-836. (10.1126/science.aad2808) 27174989

[31] Stairs S, Nikmal A, Bučar D-K, Zheng S-L, Szostak JW, Powner MW. 2017 Divergent prebiotic synthesis of pyrimidine and 8-oxo-purine ribonucleotides. Nat. Commun. **8**, 15270. (10.1038/ncomms15270) 28524845 PMC5454461

[32] Xu J et al. 2020 Selective prebiotic formation of RNA pyrimidine and DNA purine nucleosides. Nature **582**, 60-66. (10.1038/s41586-020-2330-9) 32494078 PMC7116818

[33] Powner MW, Gerland B, Sutherland JD. 2009 Synthesis of activated pyrimidine ribonucleotides in prebiotically plausible conditions. Nature **459**, 239-242. (10.1038/nature08013) 19444213

[34] Keller MA, Turchyn AV, Ralser M. 2014 Non-enzymatic glycolysis and pentose phosphate pathway-like reactions in a plausible Archean ocean. Mol. Syst. Biol. **10**, 725. (10.1002/msb.20145228) 24771084 PMC4023395

[35] Muchowska KB, Varma SJ, Moran J. 2019 Synthesis and breakdown of universal metabolic precursors promoted by iron. Nature **569**, 104-107. (10.1038/s41586-019-1151-1) 31043728 PMC6517266

[36] Krishnamurthy R, Guntha S, Eschenmoser A. 2000 Regioselective *α*-phosphorylation of aldoses in aqueous solution. Angew. Chem. Int. Ed. **39**, 2281-2285. (10.1002/1521-3773(20000703)39:13<2281::AID-ANIE2281>3.0.CO;2-2) 10941064

[37] Coggins AJ, Powner MW. 2017 Prebiotic synthesis of phosphoenol pyruvate by α-phosphorylation-controlled triose glycolysis. Nat. Chem. **9**, 310-317. (10.1038/nchem.2624) 28338685

[38] Springsteen G, Yerabolu JR, Nelson J, Rhea CJ, Krishnamurthy R. 2018 Linked cycles of oxidative decarboxylation of glyoxylate as protometabolic analogs of the citric acid cycle. Nat. Commun. **9**, 91. (10.1038/s41467-017-02591-0) 29311556 PMC5758577

[39] Trefil J, Morowitz HJ, Smith E. 2009 The origin of life: a case is made for the descent of electrons. Am. Sci. **97**, 206-213. (10.1511/2009.78.206)

[40] Schrodinger E, Penrose R. 2012 What is life?: with mind and matter and autobiographical sketches [internet]. Cambridge, UK: Cambridge University Press. [cited 24 May 2021]. See http://ebooks.cambridge.org/ref/id/CBO9781107295629.

[41] Mitchell P. 1961 Coupling of phosphorylation to electron and hydrogen transfer by a chemi-osmotic type of mechanism. Nature **191**, 144-148. (10.1038/191144a0) 13771349

[42] Martin WF, Sousa FL, Lane N. 2014 Energy at life's origin. Science **344**, 1092-1093. (10.1126/science.1251653) 24904143

[43] Todd ZR, Szabla R, Szostak JW, Sasselov DD. 2019 UV photostability of three 2-aminoazoles with key roles in prebiotic chemistry on the early earth. Chem. Commun. **55**, 10 388-10 391. (10.1039/C9CC05265H) PMC9631353 31380533

[44] Todd ZR, Fahrenbach AC, Ranjan S, Magnani CJ, Szostak JW, Sasselov DD. 2020 Ultraviolet-driven deamination of cytidine ribonucleotides under planetary conditions. Astrobiology **20**, 878-888. (10.1089/ast.2019.2182) 32267736 PMC9634989

[45] Zhu TF, Adamala K, Zhang N, Szostak JW. 2012 Photochemically driven redox chemistry induces protocell membrane pearling and division. Proc. Natl Acad. Sci. USA **109**, 9828-9832. (10.1073/pnas.1203212109) 22665773 PMC3382484

[46] Flores E, Martinez E, Rodriguez LE, Weber JM, Khodayari A, VanderVelde DG, Barge LM. 2021 Effects of amino acids on phosphate adsorption onto iron (oxy)hydroxide minerals under early earth conditions. ACS Earth Space Chem. **5**, 1048-1057. (10.1021/acsearthspacechem.1c00006)

[47] Jordan SF, Rammu H, Zheludev IN, Hartley AM, Maréchal A, Lane N. 2019 Promotion of protocell self-assembly from mixed amphiphiles at the origin of life. Nat. Ecol. Evol. **3**, 1705-1714. (10.1038/s41559-019-1015-y) 31686020

[48] Copley SD, Smith E, Morowitz HJ. 2007 The origin of the RNA world: co-evolution of genes and metabolism. Bioorg. Chem. **35**, 430-443. (10.1016/j.bioorg.2007.08.001) 17897696

[49] Lane N, Allen JF, Martin W. 2010 How did LUCA make a living? Chemiosmosis in the origin of life. Bioessays **32**, 271-280. (10.1002/bies.200900131) 20108228

[50] Pascal R, Pross A, Sutherland JD. 2013 Towards an evolutionary theory of the origin of life based on kinetics and thermodynamics. Open Biol. **3**, 130156. (10.1098/rsob.130156) 24196781 PMC3843823

[51] Pross A, Khodorkovsky V. 2004 Extending the concept of kinetic stability: toward a paradigm for life. J. Phys. Org. Chem. **17**, 312-316. (10.1002/poc.729)

[52] Pross A, Pascal R. 2017 How and why kinetics, thermodynamics, and chemistry induce the logic of biological evolution. Beilstein J. Org. Chem. **13**, 665-674. (10.3762/bjoc.13.66) 28487761 PMC5389199

[53] Grzybowski BA, Stone HA, Whitesides GM. 2000 Dynamic self-assembly of magnetized, millimetre-sized objects rotating at a liquid–air interface. Nature **405**, 1033-1036. (10.1038/35016528) 10890439

[54] Bissette AJ, Fletcher SP. 2013 Mechanisms of autocatalysis. Angew. Chem. Int. Ed. **52**, 12 800-12 826. (10.1002/anie.201303822) 24127341

[55] Blackmond DG. 2020 Autocatalytic models for the origin of biological homochirality. Chem. Rev. **120**, 4831-4847. (10.1021/acs.chemrev.9b00557) 31797671

[56] Bachmann PA, Luisi PL, Lang J. 1992 Autocatalytic self-replicating micelles as models for prebiotic structures. Nature **357**, 57-59. (10.1038/357057a0)

[57] Buhse T, Nagarajan R, Lavabre D, Micheau JC. 1997 Phase-transfer model for the dynamics of ‘micellar autocatalysis’. J. Phys. Chem. A **101**, 3910-3917. (10.1021/jp9705838)

[58] Buhse T, Lavabre D, Nagarajan R, Micheau JC. 1998 Origin of autocatalysis in the biphasic alkaline hydrolysis of C-4 to C-8 ethyl alkanoates. J. Phys. Chem. A **102**, 10 552-10 559. (10.1021/jp982765n)

[59] Adamski P, Eleveld M, Sood A, Kun Á, Szilágyi A, Czárán T, Szathmáry E, Otto S. 2020 From self-replication to replicator systems en route to de novo life. Nat. Rev. Chem. **4**, 386-403. (10.1038/s41570-020-0196-x) 37127968

[60] Carnall JMA, Waudby CA, Belenguer AM, Stuart MCA, Peyralans JJ-P, Otto S. 2010 Mechanosensitive Self-replication driven by self-organization. Science **327**, 1502-1506. (10.1126/science.1182767) 20299594

[61] Colomb-Delsuc M, Mattia E, Sadownik JW, Otto S. 2015 Exponential self-replication enabled through a fibre elongation/breakage mechanism. Nat. Commun. **6**, 7427. (10.1038/ncomms8427) 26081104 PMC4557357

[62] Otto S. 2002 Selection and amplification of hosts from dynamic combinatorial libraries of macrocyclic disulfides. Science **297**, 590-593. (10.1126/science.1072361) 12142534

[63] Xu S, Giuseppone N. 2008 Self-duplicating amplification in a dynamic combinatorial library. J. Am. Chem. Soc. **130**, 1826-1827. (10.1021/ja710248q) 18211071

[64] Cafferty BJ, Wong ASY, Semenov SN, Belding L, Gmür S, Huck WTS, Whitesides GM. 2019 Robustness, entrainment, and hybridization in dissipative molecular networks, and the origin of life. J. Am. Chem. Soc. **141**, 8289-8295. (10.1021/jacs.9b02554) 31035761

[65] Cafferty BJ, Gállego I, Chen MC, Farley KI, Eritja R, Hud NV. 2013 Efficient self-assembly in water of long noncovalent polymers by nucleobase analogues. J. Am. Chem. Soc. **135**, 2447-2450. (10.1021/ja312155v) 23394182

[66] Chen MC, Cafferty BJ, Mamajanov I, Gallego I, Krishnamurthy R, Hud NV. 2014 Spontaneous prebiotic formation of a β-ribofuranoside that self-assembles with a complementary heterocycle. J. Am. Chem. Soc. **136**, 5640-5646. (10.1021/ja410124v) 24328232

[67] von Kiedrowski G. 1993 Minimal replicator theory I: parabolic versus exponential growth. In Bioorganic chemistry frontiers (eds H Dugas, FP Schmidtchen), pp. 113-146. Berlin, Germany: Springer. See http://link.springer.com/10.1007/978-3-642-78110-0_4.

[68] Hordijk W, Vaidya N, Lehman N. 2014 Serial transfer can aid the evolution of autocatalytic sets. J. Syst. Chem. **5**, 4. (10.1186/1759-2208-5-4) 24883117 PMC4034168

[69] de Vladar HP, Santos M, Szathmáry E. 2017 Grand views of evolution. Trends Ecol. Evol. **32**, 324-334. (10.1016/j.tree.2017.01.008) 28245930

[70] Van Valen L. 1974 Molecular evolution as predicted by natural selection. J. Mol. Evol. **3**, 89-101. (10.1007/BF01796554) 4407466

[71] Eigen M. 1971 Selforganization of matter and the evolution of biological macromolecules. Naturwissenschaften **58**, 465-523. (10.1007/BF00623322) 4942363

[72] Eigen M, Schuster P. 1979 The hypercycle. Berlin, Germany: Springer. See http://link.springer.com/10.1007/978-3-642-67247-7.

[73] Monreal Santiago G, Liu K, Browne WR, Otto S. 2020 Emergence of light-driven protometabolism on recruitment of a photocatalytic cofactor by a self-replicator. Nat. Chem. **12**, 603-607. (10.1038/s41557-020-0494-4) 32591744

[74] Ottelé J, Hussain AS, Mayer C, Otto S. 2020 Chance emergence of catalytic activity and promiscuity in a self-replicator. Nat. Catal. **3**, 547-553. (10.1038/s41929-020-0463-8)

[75] Boekhoven J, Hendriksen WE, Koper GJM, Eelkema R, van Esch JH. 2015 Transient assembly of active materials fueled by a chemical reaction. Science **349**, 1075-1079. (10.1126/science.aac6103) 26339025

[76] Tena-Solsona M, Wanzke C, Riess B, Bausch AR, Boekhoven J. 2018 Self-selection of dissipative assemblies driven by primitive chemical reaction networks. Nat. Commun. **9**, 2044. (10.1038/s41467-018-04488-y) 29795292 PMC5966463

[77] Zepik HH, Blöchliger E, Luisi PL. 2001 A chemical model of homeostasis. Angew. Chem. Int. Ed. **40**, 199-202. (10.1002/1521-3773(20010105)40:1<199::AID-ANIE199>3.0.CO;2-H) 29711953

[78] Chen C, Tan J, Hsieh M-C, Pan T, Goodwin JT, Mehta AK, Grover MA, Lynn DG. 2017 Design of multi-phase dynamic chemical networks. Nat. Chem. **9**, 799-804. (10.1038/nchem.2737) 28754943

[79] Colomer I, Borissov A, Fletcher SP. 2020 Selection from a pool of self-assembling lipid replicators. Nat. Commun. **11**, 176. (10.1038/s41467-019-13903-x) 31924788 PMC6954257

[80] Moulin E, Armao JJ, Giuseppone N. 2019 Triarylamine-based supramolecular polymers: structures, dynamics, and functions. Acc. Chem. Res. **52**, 975-983. (10.1021/acs.accounts.8b00536) 30915835

[81] Rubinov B, Wagner N, Rapaport H, Ashkenasy G. 2009 Self-replicating amphiphilic β-sheet peptides. Angew. Chem. Int. Ed. **48**, 6683-6686. (10.1002/anie.200902790) 19644990

[82] Liu Z, Wu L-F, Xu J, Bonfio C, Russell DA, Sutherland JD. 2020 Harnessing chemical energy for the activation and joining of prebiotic building blocks. Nat. Chem. **12**, 1023-1028. (10.1038/s41557-020-00564-3) 33093680 PMC7610406

[83] Ricardo A, Carrigan MA, Olcott AN, Benner SA. 2004 Borate minerals stabilize ribose. Science **303**, 196. (10.1126/science.1092464) 14716004

[84] Ritson DJ, Battilocchio C, Ley SV, Sutherland JD. 2018 Mimicking the surface and prebiotic chemistry of early Earth using flow chemistry. Nat. Commun. **9**, 1821. (10.1038/s41467-018-04147-2) 29739945 PMC5940729

[85] Murillo-Sánchez S, Beaufils D, González Mañas JM, Pascal R, Ruiz-Mirazo K. 2016 Fatty acids’ double role in the prebiotic formation of a hydrophobic dipeptide. Chem. Sci. **7**, 3406-3413. (10.1039/C5SC04796J) 29997836 PMC6007129

[86] Adamala K, Szostak JW. 2013 Competition between model protocells driven by an encapsulated catalyst. Nat. Chem. **5**, 495-501. (10.1038/nchem.1650) 23695631 PMC4041014

[87] Chen IA, Roberts RW, Szostak JW. 2004 The emergence of competition between model protocells. Science **305**, 1474-1476. (10.1126/science.1100757) 15353806 PMC4484590

[88] Piedrafita G, Monnard P-A, Mavelli F, Ruiz-Mirazo K. 2017 Permeability-driven selection in a semi-empirical protocell model: the roots of prebiotic systems evolution. Sci. Rep. **7**, 3141. (10.1038/s41598-017-02799-6) 28600550 PMC5466667

[89] Bonfio C, Valer L, Scintilla S, Shah S, Evans DJ, Jin L, Szostak JW, Sasselov DD, Sutherland JD, Mansy SS. 2017 UV-light-driven prebiotic synthesis of iron–sulfur clusters. Nat. Chem. **9**, 1229-1234. (10.1038/nchem.2817) 29168482 PMC5808832

[90] Bonfio C, Godino E, Corsini M, de Biani F F, Guella G, Mansy SS. 2018 Prebiotic iron–sulfur peptide catalysts generate a pH gradient across model membranes of late protocells. Nat. Catal. **1**, 616-623. (10.1038/s41929-018-0116-3)

[91] Basak S, Nader S, Mansy SS. 2021 Protometabolic reduction of NAD+ with α-Keto acids. JACS Au **1**, 371-374. (10.1021/jacsau.0c00124) 34467301 PMC8395669

